# Effect of a Liver Cancer Education Program on Hepatitis B Screening Among Asian Americans in the Baltimore–Washington Metropolitan Area, 2009–2010

**DOI:** 10.5888/pcd11.130258

**Published:** 2014-02-06

**Authors:** Hee-Soon Juon, Sunmin Lee, Carol Strong, Rajiv Rimal, Gregory D Kirk, Janice Bowie

**Affiliations:** Author Affiliations: Sunmin Lee, University of Maryland School of Public Health, College Park, Maryland; Carol Strong, National Cheng Kung University, Tainan City, Taiwan; Rajiv Rimal, George Washington University, Washington, DC; Gregory D Kirk, Janice Bowie, Johns Hopkins University, Baltimore, Maryland.

## Abstract

**Introduction:**

Asian Americans have the highest incidence of hepatocellular carcinoma (HCC), the major form of primary liver cancer, of all ethnic groups in the United States. Chronic hepatitis B virus (HBV) infection is the most common cause of HCC, and as many as 1 in 10 foreign-born Asian Americans are chronically infected with HBV. We tested the effectiveness of a culturally tailored liver cancer education program for increasing screening for HBV among Chinese, Korean, and Vietnamese Americans residing in the Baltimore–Washington metropolitan area, from November 2009 through June 2010.

**Methods:**

We used a cluster randomized controlled trial to recruit volunteer participants from community-based organizations (CBOs) in the Baltimore–Washington metropolitan area. We selected 877 participants by using a pretest survey. People were eligible to participate if they had not attended a hepatitis B–related education program in the past 5 years. The intervention group (n = 441) received a 30-minute educational program, and the control group (n = 436) received an educational brochure. After attending the educational program, the intervention group completed a post-education survey. Six months later, participants in both groups were followed up by telephone. Receipt of HBV screening was the outcome measure.

**Results:**

Approximately 79% (n = 688) of participants completed the 6-month follow-up telephone survey. Among those who had not had HBV screening at baseline (n = 446), the adjusted odds of self-reported receipt of HBV screening at the 6-month follow-up to the educational program were significantly higher for the intervention group than for the control group (odds ratio = 5.13; 95% confidence interval, 3.14–8.39; *P* < .001). Chinese Americans and Vietnamese Americans had significantly higher odds of having HBV screening in the 6-month period than Korean Americans.

**Conclusion:**

Culturally tailored education programs that increase liver cancer awareness can be effective in increasing HBV screening among underserved Asian American populations.

## Introduction

Cancer is the leading cause of death among Asian Americans (1). Although progress has been made in lowering cancer incidence and mortality, many minority groups have not fully or equitably benefited from this progress (2). Hepatocellular carcinoma (HCC) is the major form of primary liver cancer. Asian Americans have the highest HCC incidence and mortality rates of all ethnic groups in the United States (3). Chronic hepatitis B virus (HBV) infection is the most common cause of HCC, accounting for 80% of all cases worldwide (4). Although HBV prevalence is low among whites in the United States (0.1%), as many as 1 in 10 foreign-born Asian Americans are chronically infected with HBV (5). The prevalence of chronic HBV among Asian immigrants to the United States is generally reflective of the HBV prevalence in their countries of origin and varies from 2% in Japanese Americans to 11.3% in Chinese Americans (6). In a recent study of Asian Americans in Maryland, 4.5% were chronically infected with HBV (7). A study in New York City reported prevalence of chronic HBV infection among Asian Americans in that city at 14.8% (8).

HCC (hereafter referred to as “liver cancer”) incidence and mortality rates are higher among Asian Americans than any other ethnic group in the United States (9). A quarter of patients with HBV will eventually die of liver cancer or liver failure (10). Despite existing public health efforts on HBV vaccination, liver cancer incidence among Asian Americans increased from 1990 to 2008 (11). In a recent study of age-adjusted cancer rates among Asian Americans in California (12), Vietnamese men had the highest incidence of liver cancer of all racial/ethnic groups (54.3 per 100,000). Incidence among Korean men (33.7 per 100,000) and Chinese men (23.3 per 100,000) was much higher than among non-Hispanic white men (6.8 per 100,000).

Our study, the Asian American Liver Cancer Education Program, was conducted in response to this cancer-related health disparity. The program’s goal is to develop and implement a culturally integrated educational program to promote HBV screening and increase awareness of liver cancer prevention among the high-risk Asian American population. Because many Asian Americans are not acculturated and are often linguistically isolated, they are considered a hard-to-reach population for the development and implementation of cancer education and prevention programs. We conducted a randomized controlled study to evaluate the effectiveness of a liver cancer education program focusing on HBV screening behavior among 3 Asian American subgroups: Chinese, Korean, and Vietnamese.

## Methods

### Overview of study design

We used a cluster randomized control design to recruit Asian Americans aged 18 years or older from the membership of Asian community-based organizations (CBOs) in the Baltimore–Washington metropolitan area. Asian communities are closely connected in their respective ethnic subgroups because they go to the same churches and send their children to the same language schools in the counties where they reside. We were aware of the possibility that participants in our intervention group might share information from our educational program with our control group; to avoid contamination between intervention and control groups, we chose different counties for the 2 groups, a method used in a previous study (13). In our study, the Baltimore metropolitan area (ie, Baltimore City, Baltimore County, and Anne Arundel County) was selected as the intervention site, and the Washington, DC–suburban Maryland area (ie, Montgomery County and Prince George’s County) was selected as the control site. Each CBO was randomly selected from either an intervention or control site. A total of 27 CBOs, 15 in the intervention site and 12 in the control site, agreed to participate in our study. Nine of these organizations served Korean Americans, 8 served Chinese Americans, and 10 served Vietnamese Americans. People were eligible to participate if they had not attended a hepatitis B-related education program in the past 5 years. Participants that met the study’s eligibility criterion were then recruited from among members of these organizations on a voluntary basis. Korean participants came predominantly from churches (n = 7), which made up 70.0% of the organizations for the Korean recruitment. Chinese participants were enrolled predominantly from language schools (n = 5), which made up 63% of participating Chinese organizations. Vietnamese participants did not have a predominant recruitment site, and study participants were recruited from various venues such as temples, churches, and nail salons (14–16). This study protocol was approved by the institutional review board of the Johns Hopkins Bloomberg School of Public Health (IRB study no. 00001622; approval date, October 1, 2009).

The Figure summarizes the study design. Of the 940 eligible volunteer participants from the 27 CBOs, 5.0% (n = 47) did not show up for the program. Of the 893 who came to the educational program, 13 did not complete a baseline survey or did not participate in the program, and 3 had participated in a liver education program in the past year. Of the 877 participants who completed the baseline survey, 441 (50.3%) drawn from 15 CBOs were in the intervention group and 436 (49.7%) drawn from 12 CBOs were in the control group.

**Figure:**
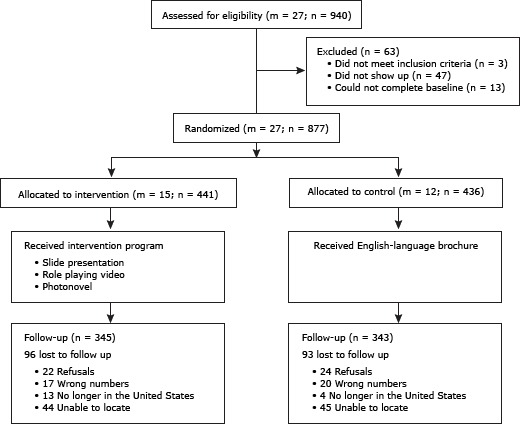
Flow of participants through a randomized controlled trial in a study of the effect of a liver cancer education program on hepatitis B screening among Chinese, Korean, and Vietnamese Americans (N = 877) in the Baltimore–Washington Metropolitan Area, 2009–2010. In equations, m refers to number of clusters (community-based organizations) and n refers to number of participants.

### Data collection procedures

From November 2009 through June 2010, self-identified Asian American adults in the Baltimore–Washington metropolitan area were recruited from various Chinese, Korean, and Vietnamese CBOs, such as churches, temples, language schools, and college cultural organizations. Asian grocery stores, restaurants, and nail salons were additional sources for recruitment. Membership in a CBO was not required for participation. We excluded from our study people who had attended a previous educational program on HBV infection and liver cancer prevention (15).

Participants in both the intervention and control groups completed a self-administered paper survey before they received the educational program. Surveys were translated into Chinese, Korean, and Vietnamese. Trained bilingual research assistants assisted participants who had reading problems (16). Participants in the intervention group received a culturally integrated 30-minute liver cancer education program. Those in the control group received the English-language brochure, *What I Need to Know About Hepatitis B,* developed by the National Institute of Diabetes and Digestive and Kidney Diseases. After 6 months, all participants were contacted via telephone for a follow-up interview asking if they had been screened for HBV. We delivered the intervention program to participants in the control group after the final follow-up interview.

### Intervention

The intervention arm of the study was developed using the PRECEDE–PROCEED planning model, which helps to identify factors affecting health behavior and to develop an educational message (17). This model was used as a guide for understanding the needs of target populations; planning the intervention; program development, implementation, and evaluation (process, impact, and outcome); policy implications; and obtaining feedback (17). We also incorporated findings from focus groups conducted by Philbin et al to accommodate traditional and cultural beliefs of Asian Americans (18). These focus groups had been conducted in the preliminary phase of our study to identify participants’ level of knowledge and barriers related to screening for each ethnic group (18). We then consulted a community advisory board (CAB) made up of local leaders from the participating ethnic groups to obtain their feedback on the overall program. CAB members served as the “voice of the community” and also advised program staff on the intervention. They also kept research staff and other CAB members abreast of projects and activities conducted in the community.

Our education program for the intervention group was a 30-minute educational session consisting of 3 parts: 1) a slide presentation of comprehensive information about HBV by trained bilingual staff, 2) a role-play video that showed ideal patient–physician communication at the clinic, and 3) an ethnicity-specific photonovel (19). Materials featured Asian Americans.

### Outcome measures

The primary outcome measure was whether participants who were unscreened at baseline (the pretest survey) reported having had an HBV screening test in the 6 months following the intervention.

We collected information on sociodemographic characteristics and family history of HBV infection for participants in the control and intervention groups at baseline. Age, sex, education, employment, race/ethnicity, proportion of life spent in the United States, health insurance status, self-rated physical health, depressive symptoms, and family history of HBV infection were compared between the intervention and the control groups.

We compared baseline sociodemographic characteristics of the intervention and control groups to show the result of random assignment at the individual and cluster (CBO) level, as suggested by the Consort statement for cluster randomized trials (20). For the primary outcome, receipt of HBV screening within 6 months of receiving the educational program, we compared the proportion of participants in the intervention group who received screening with the proportion in the control group who were screened among those who completed the posttest (the 6-month follow-up phone call) and who did not report having had HBV screening in the baseline survey (n = 446). We used multiple logistic regression analysis with generalized estimating equation to adjust for the cluster effect. In addition to adjusting for the intervention effect, we further adjusted for ethnicity to show the variation of screening behavior within 6 months among the 3 ethnic groups. We used STATA version 11 (Stata Corp, College Station, Texas) for all analyses.

## Results

Approximately 78.0% of participants (n = 688) completed the telephone survey at the 6-month follow-up. Those who dropped out of the study (n = 189; attrition rate, 21.6%) were not statistically different from those who remained in both arms in age, sex, marital status, family history of HBV infection, health status, baseline HBV screening, and knowledge of HBV infection. However, education was associated with dropouts. College graduates were more likely to follow-up than their less-educated counterparts (57.4% vs 37.6%, *P* < .001). Race/ethnicity was also associated with dropping out; Vietnamese were more likely to drop out than Chinese or Koreans (39.7%, 34.9%, and 25.4%, respectively, *P* .010). We reached 78.2% of intervention group participants and 78.7% of control group participants for telephone follow-up.

At baseline, the average age of the 877 participants was 45.1 years (standard deviation [SD], 13.5 y); 58.5% were women and 13.2% had less than a high school education Table 1. Approximately two-thirds of participants had health insurance. About 14% reported that they had a family history of HBV infection. About 38% had spent less than 25% of their lives in the United States. The 2 study groups appeared well-matched at both the individual and cluster levels with respect to demographic characteristics, socioeconomic status, health insurance coverage, and self-rated physical and mental health.

**Table 1:** Baseline Participant Characteristics at Individual and Cluster Levels, Study of the Effect of a Liver Cancer Education Program on Hepatitis B Screening Among Chinese, Korean, and Vietnamese Americans (N = 877) in the Baltimore–Washington Metropolitan Area, 2009–2010

Characteristic	Total (n = 877)	Intervention (n = 441)	Control, (n = 436)
**Individual level**			
**Age, y, mean (SD)**	45.1 (13.5)	46.9 (12.7)	43.3 (14.0)
**Sex, %**			
Female	58.5	57.6	59.4
Male	41.5	42.4	40.6
**Ethnicity^a^, %**			
Korean	33.5	33.6	33.5
Vietnamese	31.9	32.2	31.7
Chinese	34.5	34.2	34.9
**Education, %**			
Less than high school	13.2	13.6	12.8
High school graduate or some college	33.6	31.1	36.2
College graduate	53.1	55.3	50.9
**Employed, %**	60.5	62.0	58.9
**Spent less than 25% of life in United States, %**	37.9	39.9	35.9
**Has health insurance, %**	66.2	68.0	64.4
**Family history of hepatitis B infection, %**	14.4	15.5	13.4
**Self-rated health^b^, mean (SD)**	3.04 (1.05)	3.09 (1.04)	3.00 (1.06)
**Depressed (CES-D >16), %**	25.5	26.1	24.9
**Cluster level**_,_ **mean (SD)^c^**			
**Age, y**	41.8 (8.9)	43.6 (7.9)	39.5 (9.8)
**Sex, %**			
Female	19.0 (14.4)	16.9 (11.2)	21.6 (17.7)
Male	13.5 (10.2)	12.5 (8.7)	14.8 (12.1)
**Education**			
Less than high school	4.3 (4.2)	4.0 (4.6)	4.7 (3.8)
High school graduate/some college	10.9 (9.5)	9.1 (8.8)	13.2 (10.2)
College graduate	17.3 (15.8)	16.3 (11.7)	18.5 (20.3)
**Employed**	19.6 (14.7)	18.2 (11.7)	21.3 (18.2)
**Spent less than 25% of life in US, %**	12.3 (9.5)	11.7 (7.9)	13.0 (11.5)
**Has health insurance**	21.5 (17.2)	20.0 (14.4)	23.4 (20.7)
**Family history of hepatitis B infection**	4.7 (4.0)	4.5 (4.0)	4.8 (4.2)
**Self-rated health (** **1** **–** **5** **)^b^**	2.98 (0.45)	2.97 (0.45)	2.99 (0.48)
**Depressed (CES-D >16)**	8.3 (6.7)	7.7 (6.4)	9.0 (7.3)

Abbreviations: SD, standard deviation; CES-D, Center for Epidemiologic Studies Depression Scale (31).

a Self-identified as Asian based on country of birth and language.

b Reported on a scale of 1 to 5 (1 = excellent, 2 = very good , 3 = good , 4 = fair , 5 = poor).

c Based on number of participants from each cluster (community-based organization) unless otherwise indicated.

Table 2 presents the descriptive statistics and adjusted odds ratio for the primary study outcome, the number of participants not previously tested (n = 446) who reported they had been tested for HBV at 6-month follow-up, for intervention and control groups. A total of 27 clusters (CBOs) were included in the study, 15 for the intervention group (n = 220) and 12 for the control group (n = 226). The intervention group had higher HBV screening rates at follow-up than the control group (33.6% vs 9.7%) without any adjustment.

**Table 2:** Multiple Logistic Regression Analysis of Hepatitis B Screening Behavior at 6-Month Follow-up Among Previously Unscreened Participants (n = 446), Study of the Effect of a Liver Cancer Education Program on Hepatitis B Screening Among Chinese, Korean, and Vietnamese Americans in the Baltimore–Washington Metropolitan Area, 2009–2010

Variable	Total	Intervention group	Control group
Number of clusters	27	15	12
Number of participants	446	220	226
Percentage screened for hepatitis B	21.5	33.6	9.7

**Intervention effect**	**Adjusted Odds Ratio** **(95% Confidence Interval)^a^**		***P* Value^a^**

**Model 1^b^**			
Control group	1 [Reference]		<.001
Received intervention	4.63 (2.30–9.32)		
**Model 2^b^**			
Control group	1 [Reference]		<.001
Received intervention	5.13 (3.14–8.39)		
**Ethnicity**			
Korean	1 [Reference]		<.001
Chinese (vs Korean)	3.00 (1.66–5.41)		
Vietnamese (vs Korean)	3.59 (2.04–6.34)		

a Odds ratios, 95% confidence intervals, and *P* values were obtained from multiple logistic regression models comparing intervention and control groups adjusted for age and clustering within randomized groups for both models.

b Calculated by using generalized estimating equation. Model 1 used generalized estimating equation to adjust for the cluster effect and age, and Model 2 further adjusted for ethnicity.

In the multiple logistic regression analysis, the overall adjusted odds of a participant self-reporting having had HBV screening at 6-month follow-up was significantly higher for the intervention group than for the control group (odds ratio [OR], 4.63; 95% confidence interval [CI], 2.30–9.32, *P* < .001; [Table 2]). When further adjusted for ethnicity in Model 2, the odds of HBV screening at 6-month follow-up for the intervention group increased to 5.13 compared with the control group (95% CI, 3.14–8.39; *P* < .001). We also found that Chinese Americans and Vietnamese Americans had higher odds of being screened at 6-month follow-up than Korean Americans, after adjusting for the intervention effect (OR, 3.00; 95% CI, 1.66–5.41 for Chinese Americans and OR, 3.59; 95% CI, 2.04–6.34 for Vietnamese Americans; both *P* < .001). Several factors motivated participants in the intervention group to obtain HBV screening in the 6 months following the educational program (Table 3). Among the 74 respondents in the intervention group who obtained HBV screening in that period, 78.4% (n = 58) reported that our educational program motivated them to do so. In the subset analysis of the 58 who were motivated by our education program, 86.2% identified the slide presentation as the primary motivator followed by the role-playing video (32.8%) and photonovel (31.0%). Discussion after the presentation (25.9%) and the resource list for HBV screening (15.5%) were also important motivators for screening.

**Table 3:** Motivating Factors for Obtaining Hepatitis B Screening Among Participants in the Intervention Group (n = 74), Study of the Effect of a Liver Cancer Education Program on Hepatitis B Screening Among Chinese, Korean, and Vietnamese Americans in the Baltimore–Washington Metropolitan Area, 2009–2010

Motivating Factors for Hepatitis B Screening (n = 74)	n (%)^a^
Our intervention program	58 (78.4)
Self-awareness	11 (14.9)
Annual check-up	6 (8.1)
Free screening event	6 (8.1)
Physician’s recommendation	5 (6.8)
Work requirement	2 (2.7)
**Most motivating components of our intervention program** (n = 58)	
Educational slide presentation	50 (86.2)
Role playing video	19 (32.8)
Photonovel	18 (31.0)
Discussion/questions and answers after presentation	15 (25.9)
List of resources of free and low-cost hepatitis B screening	9 (15.5)

a Total exceeds 100% because multiple responses were allowed.

## Discussion

Liver cancer is one of the few cancers with increasing incidence rates in the United States (21). This is due in part to high HBV infection rates among foreign-born Asian Americans (9). Moreover, chronic HBV infection is often asymptomatic. People who are infected can appear and feel healthy but can still transmit the virus, which can advance to severe liver diseases. HBV screening is the primary liver cancer prevention method; it enables identification of persons at risk to encourage them to obtain a series of HBV vaccinations as the secondary prevention method. All these preventive practices begin with identification of a person’s status of HBV infection. Hence, it is important to promote HBV screening in high-risk populations.

This randomized controlled trial demonstrated that a culturally integrated intervention with multiple components was effective in increasing HBV screening. We found that the odds for completing HBV screening were more than 5 times higher for the intervention group than for the control group after adjusting for ethnicity, age, and the cluster effect. This result was consistent across subgroups, although the odds ratio was highest for the Vietnamese intervention group and lowest for the Korean intervention group. Consistent with previous studies in HBV testing and Papanicolaou testing among Asian Americans, the overall magnitude and consistency of findings across the subgroups strengthen confidence in the effect of the intervention (22,23).

A salient issue for being screened for HBV during the 6-month follow-up period was whether participants were motivated by our program. We learned that our culturally and linguistically tailored intervention increases HBV screening and is important for immigrants with language barriers or of lower socioeconomic status (24–27). Lower neighborhood socioeconomic status is associated with liver cancer incidence (28). Foreign-born Asian Americans are 5 times more likely to have liver cancer than their US-born counterparts (29). Promising results from our study suggest that an appropriate slide presentation may be used alone or in combination with a role-play video or photonovel in various Asian American communities to increase HBV screening. Furthermore, findings from our process evaluation of the photonovels indicate that they are effective teaching tools, especially for less educated and low-literacy participants (19).

Our study has some limitations. First, the primary outcome, receipt of HBV screening, was self-reported. Some participants were confused about the difference between a liver function test and an HBV screening test. We clarified these 2 tests in the survey, pointing out that the HBV screening test is only required once in a lifetime. Nevertheless, the extent of reliability and validity of the self-report is not certain. In a study that evaluated a hepatitis intervention, Taylor et al reported low cross-verification of self-reports of screening with the medical record (22). Future study is recommended that includes HBV serological testing to assess the status of HBV infection. Second, findings in these concentrated populations in the Baltimore–Washington metropolitan area may not be generalizable to Asians who are more acculturated or living in other types of communities or geographical settings. Third, those who voluntarily participated in the study might be more motivated to learn about HBV infection and liver cancer, and these findings could overestimate the effect of a similar intervention if conducted within a less motivated population. However, the use of a similarly motivated control group that was recruited in the same manner strengthens the causal validity of our intervention effect. Finally, although effective, our screening population was small and additional components or repeated interventions are likely to be needed.

Despite the limitations, a strength of this study is that culturally and linguistically integrated methods such as recruitment, face-to-face interviews, and telephone contacts built trust and rapport with participants. The intervention program, which included an HBV slide presentation, role-playing video, and photonovels, was developed and tested in respective Asian languages with pictures featuring Asian Americans. This likely helped participants identify with messages in the materials. Because participants were interacting with researchers, staff, and interviewers with the same ethnic background, they likely felt more comfortable with asking questions in their languages. Conducting training in recipients’ native language may be an effective tool to motivate them to have screening and to increase knowledge of HBV infection. Second, we were successful in recruiting hard-to-reach and vulnerable populations in spite of challenges in capturing these groups, which face cultural, linguistic, and economic barriers. CAB members helped us to determine how and where to recruit non-English speaking immigrants. CAB members shared perspectives from the community relevant to the development, implementation, and potential sustainability of effective intervention activities and used their personal and social networks to encourage the participation of CBOs and individuals. They worked closely with the research team on all aspects of the study and were essential to this research project for ensuring the cultural relevance of intervention strategies and facilitating community competence.

In conclusion, this culturally integrated intervention program yielded a substantial increase in screening behavior in underserved, high-risk, Asian minority populations. We recommend providing culturally and linguistically integrated educational programs as one way to change social norms to increase preventive behavior. These study findings provide a framework for large-scale community-based interventions to promote liver cancer prevention and control in high risk subgroups of Asian Americans, which could contribute toward achieving the *Healthy People 2020* program goal of improving HBV screening among minority populations (30).

## References

[1] National Center for Health Statistics. Health, United States, 2010: with special feature on death and dying (Table 26). http://www.cdc.gov/nchs/data/hus/hus10.pdf#026. Accessed June 15, 2012. 21634072

[2] Freeman HP . Poverty, culture, and social injustice: determinants of cancer disparities. CA Cancer J Clin 2004;54(2):72–7. 10.3322/canjclin.54.2.72 15061597

[3] National Cancer Institute. SEER cancer statistics review 2002–2006. US and SEER death rates by primary cancer site and race/ethnicity, 2002–2006 (Table 1.20). http://seer.cancer.gov/csr/1975_2006/results_single/sect_01_table.20_2pgs.pdf. Accessed June 15, 2010.

[4] Bosch FX , Ribes J , Diaz M , Cleries R . Primary liver cancer: worldwide incidence and trends. Gastroenterology 2004;127(5, Suppl 1):S5–16. 10.1053/j.gastro.2004.09.011 15508102

[5] Kim WR . Epidemiology of hepatitis B in the United States. Hepatology 2009;49(5, Suppl):S28–34. 10.1002/hep.22975 19399791 PMC3290915

[6] Hu KQ . Hepatitis B virus (HBV) infection in Asian and Pacific Islander Americans (APIAs): how can we do better for this special population? Am J Gastroenterol 2008;103(7):1824–33. 10.1111/j.1572-0241.2008.01878.x 18479498

[7] Hsu CE , Liu LC , Juon HS , Chiu YW , Bawa J , Tillman U , Reducing liver cancer disparities: a community-based hepatitis-B prevention program for Asian-American communities. J Natl Med Assoc 2007;99(8):900–7. 17722668 PMC2574302

[8] Centers for Disease Control and Prevention. Screening for chronic hepatitis B among Asian/Pacific Islander populations–New York City, 2005. MMWR Morb Mortal Wkly Rep 2006;55(18):505–9. 16691180

[9] Altekruse SF , McGlynn KA , Reichman ME . Hepatocellular carcinoma incidence, mortality, and survival trends in the United States from 1975 to 2005. J Clin Oncol 2009;27(9):1485–91. 10.1200/JCO.2008.20.7753 19224838 PMC2668555

[10] Beasley RP . Hepatitis B virus. The major etiology of hepatocellular carcinoma. Cancer 1988;61(10):1942–56. 10.1002/1097-0142(19880515)61:10<1942::AID-CNCR2820611003>3.0.CO;2-J 2834034

[11] Gomez SL , Noone AM , Lichtensztajn DY , Scoppa S , Gibson JT , Liu L , Cancer incidence trends among Asian American populations in the United States, 1990–2008. J Natl Cancer Inst 2013;105(15):1096–110. 10.1093/jnci/djt157 23878350 PMC3735462

[12] McCracken M , Olsen M , Chen MS Jr , Jemal A , Thun M , Cokkinides V , Cancer incidence, mortality, and associated risk factors among Asian Americans of Chinese, Filipino, Vietnamese, Korean, and Japanese ethnicities. CA Cancer J Clin 2007;57(4):190–205. 10.3322/canjclin.57.4.190 17626117

[13] Juon HS . Seung-Lee C , Klassen AC . Predictors of regular Pap smears among Korean-American women. Prev Med 2003;37(6 Pt 1):585–92. 10.1016/j.ypmed.2003.09.006 14636792

[14] Juon HS , Park BJ . Effectiveness of a culturally integrated liver cancer education in improving HBV knowledge among Asian Americans. Prev Med 2013;56(1):53–8. 10.1016/j.ypmed.2012.11.003 23159302 PMC3540148

[15] Chen L , Juon HS , Lee S . Acculturation and BMI among Chinese, Korean, and Vietnamese adults. J Community Health 2012;37(3):539–46. 10.1007/s10900-011-9476-1 21922164 PMC3804273

[16] Strong C , Lee S , Tanaka M , Juon HS . Ethnic differences in prevalence and barriers of HBV screening and vaccination among Asian Americans. J Community Health 2012;37(5):1071–80. 10.1007/s10900-012-9541-4 22302652 PMC3804552

[17] Green LW , Kreuter MW . Health promotion planning: an educational and environmental approach. Mountain View (CA): Mayfield; 1991.

[18] Philbin MM , Erby LA , Lee S , Juon HS . Hepatitis B and liver cancer among three Asian American sub-groups: a focus group inquiry. J Immigr Minor Health 2012;14(5):858–68. 10.1007/s10903-011-9523-0 21901445 PMC3804298

[19] Lee S , Yoon H , Chen L , Juon HS . Culturally appropriate photonovel development and process evaluation for hepatitis B prevention in Chinese, Korean, and Vietnamese American communities. Health Educ Behav 2013; 40(6):694-703. 10.1177/1090198112474003 23372031 PMC3830675

[20] Campbell MK , Piaggio G , Elbourne DR , Altman DG . CONSORT Group. Consort 2010 statement: extension to cluster randomised trials. BMJ 2012;345:e5661. 10.1136/bmj.e5661 22951546

[21] El-Serag HB . Epidemiology of viral hepatitis and hepatocellular carcinoma. Gastroenterology 2012;142(6):1264–73.e1. 10.1053/j.gastro.2011.12.061 PMC3338949 22537432

[22] Taylor VM , Hislop TG , Tu SP , Teh C , Acorda E , Yip MP , Evaluation of a hepatitis B lay health worker intervention for Chinese Americans and Canadians. J Community Health 2009;34(3):165–72. 10.1007/s10900-008-9138-0 19127416 PMC2735565

[23] Centers for Disease Control and Prevention. Hepatitis B information for health professionals: transmission, symptoms, and treatment. http://www.cdc.gov/hepatitis/hbv/index.htm. Accessed May 20, 2013.

[24] Hislop TG , Teh C , Low A , Tu SP , Yasui Y , Coronado GD , Predisposing, reinforcing and enabling factors associated with hepatitis B testing in Chinese Canadians in British Columbia. Asian Pac J Cancer Prev 2007;8(1):39–44. 17477769

[25] Hislop TG , Bajdik CD , Teh C , Lam W , Tu SP , Yasui Y , Hepatitis B testing and vaccination in immigrants attending English as a second language classes in British Columbia, Canada. Asian Pac J Cancer Prev 2009;10(6):997–1002. 20192572 PMC2862471

[26] Tu R SP , Li L , Tsai JH , Yip MP , Terasaki G , Teh C , . A cross-border comparison of hepatitis B testing among Chinese residing in Canada and the United States. Asian Pac J Cancer Prev 2009;10(3):483–90. 19640196 PMC3856895

[27] Lutgehetmann M , Meyer F , Volz T , Lohse AW , Fischer C , Dandri M , Knowledge about HBV, prevention behaviour and treatment adherence of patients with chronic hepatitis B in a large referral centre in Germany. Z Gastroenterol 2010;48(9):1126–32. 10.1055/s-0029-1245304 20839162

[28] Shebl FM , Capo-Ramos DE , Graubard BI , McGlynn KA , Altekruse SF . Socioeconomic status and hepatocellular carcinoma in the United States. Cancer Epidemiol Biomarkers Prev 2012;21(8):1330–5. 10.1158/1055-9965.EPI-12-0124 22669949 PMC3647693

[29] Chang ET , Yang J , Alfaro-Velcamp T , So SKS , Glaser SL , Gomez SL . Disparities in liver cancer incidence by nativity, acculturation, and socioeconomic status in California Hispanics and Asians. Cancer Epidemiol Biomarkers Prev 2010;19(12):3106–18. 10.1158/1055-9965.EPI-10-0863 20940276 PMC3005535

[30] US Department of Health and Human Services. Healthy people 2020. http://www.healthypeople.gov/2020/topicsobjectives2020/overview.aspx?topicid=23. Accessed October 16, 2013. 10.3109/15360288.2015.1037530 26095483

[31] Radloff LS . The CES-D scale: a self-report depression scale for research in the general population. Appl Psychol Meas 1977;1(3):385–401. 10.1177/014662167700100306

